# Genome-Wide Identification, Characterization, and Expression Analysis of *NRT* Gene Family in *Suaeda glauca*

**DOI:** 10.3390/biology14081097

**Published:** 2025-08-21

**Authors:** Zitong Ou, Jin Sun, Xueli Li, Haoran Feng, Xingguang Chen, Sisi Liang, Zhonghua Guo, Lulu Wang, Xiaoping Niu, Jinbiao Ma, Sheng Wang, Yuan Qin, Yan Cheng

**Affiliations:** 1 Fujian Provincial Key Laboratory of Haixia Applied Plant Systems Biology, State Key Laboratory of Ecological Pest Control for Fujian and Taiwan Crops, College of Life Sciences, College of Plant Protection, School of Future Technology, Haixai Institute of Science and Technology, Fujian Agriculture and Forestry University, Fuzhou 350002, China; ouzitong@foxmail.com (Z.O.); sunjin0071@163.com (J.S.); 15285122994@163.com (X.L.); fhrone@163.com (H.F.); chen_xg0722@163.com (X.C.); liangsisi_2204@163.com (S.L.); hh18655786652@163.com (Z.G.); luluwanghn@163.com (L.W.); xpniu0613@126.com (X.N.); 2 Key Laboratory of Biogeography and Bioresources in Arid Land, Xinjiang Institute of Ecology and Geography, Urumqi 830011, China; majinbiao@ms.xjb.ac.cn; 3 Department of Biochemistry, Microbiology and Immunology, University of Saskatchewan, Saskatoon, SK S7N 5E5, Canada; sheng.wang@gifs.ca

**Keywords:** soil salinization, *Suaeda glauca*, *NRT* gene family, bioinformatics, gene expression analysis

## Abstract

Nitrogen (N) is crucial for plant growth and stress resistance and is primarily absorbed and transported by nitrate transporters (NRT). *Suaeda glauca*, known for its strong salt-alkali stress resistance, and *SgNRT* genes have rarely been reported. This study aims to identify and analyze the *SgNRT* gene family to understand its composition, evolutionary patterns, and roles in salt stress responses. We identified 212 SgNRTs, which were categorized into three branches, with *SgNRT1/SgNPF* and *SgNRT2* as the major families. Structural analysis, conserved domains, chromosomal localization, and collinearity were also examined. Spatiotemporal expression characteristics of *SgNRT* genes were analyzed, revealing specific expression across 13 organs or tissues and dynamic responses to salt treatment over 48 h. Notably, *SgNRT1.185*, *SgNRT2.25,* and *SgNRT2.2* exhibited rapid salt induction in leaves (activated within 0.5 h, peaking at 2 h), with *SgNRT1.185* showing relatively high upregulation. *SgNRT1.185* and *SgNRT2.35* were induced by high salt concentrations (200 mM) in both roots and leaves. *SgNRT2.35* exhibited higher basal and stress-induced levels than the other genes. Bioinformatics analysis suggests spatially specific expression of *SgNRT* genes, potentially involved in nitrogen absorption and transport across various developmental stages and organs/tissues of *Suaeda glauca*. These findings offer a theoretical basis for understanding the adaptive strategies of *Suaeda glauca* under saline-alkali stress and provide insights into the functional evolution of plant *NRT* genes, aiding in the development of stress-resistant crops.

## Simple Summary

This study comprehensively characterizes the *NRT* gene family in the halophyte *Suaeda glauca*, identifying 212 members divided into two main families, SgNRT1/SgNPF and SgNRT2. Candidate genes such as *SgNRT1.185*, *SgNRT2.2*, *SgNRT2.25*, and *SgNRT2.35* respond to salt stress, with *SgNRT2.25* and *SgNRT2.2* rapidly upregulated in leaves, *SgNRT1.185* and *SgNRT2.35* induced both in leaves and roots. *SgNRT2.35* shows high basal and salt-induced expression in roots and maintains a high expression level within 24 h. These findings suggest these genes may play a central role in nitrogen uptake and contribute to crop resilience against saline conditions, providing valuable genetic resources for developing stress-resistant crops.

## 1. Introduction

Nitrogen (N) is an essential macronutrient for plant growth and development and affects crop productivity. Plants absorb nitrogen primarily in two forms: inorganic nitrogen (nitrate) and organic nitrogen (ammonium, amino acids, and urea). Nitrate is the preferred nitrogen source for higher plants. It can be stored in vacuoles and recycled as needed [1]. Nitrate acts as a signaling molecule to break seed dormancy, regulate lateral root development, leaf growth, and flowering time, and induce genes related to plant growth and development [2,3,4]. Plant nitrate transporters comprise three major families: NRT1/PTR (also known as NPF, NITRATE TRANSPORTER 1/PEPTIDE TRANSPORTER FAMILY), NRT2, and NRT3/NAR2. Based on sequence and functional similarities, NRT1/PTR are now classified as a single family [5,6]. In *Arabidopsis*, NRT1.1 functions as a dual-affinity nitrate transporter, while other members of the NPF family exhibit broad substrate specificity. This family comprises eight subfamilies that transport not only nitrate but also nitrate/auxin, nitrate/ABA, nitrate/glucosinolates, or GA/JA [7,8,9]. NRT2 belongs to the major facilitator superfamily (MFS), and its members are high-affinity nitrate transporters [10]. In the roots of various higher plants, NRT2 requires interaction with another protein to take up nitrate, and this auxiliary protein is NRT3 [11].

To date, many NRT family members have been identified as high-affinity transport systems (HATS) and low-affinity transport systems (LATS), playing a significant role in these processes [12]. Studies have shown that plant absorption, transport, and storage of nitrate depend on the concentration of nitrate in the soil [13]. When the external nitrate concentration in the soil is low (<1 mM), HATS is beneficial for the absorption and transport of nitrate. LATS functions when the external nitrate concentration is high (>1 mM). Additionally, existing studies have revealed that nitrogen uptake plays an important role in the regulation of plant stress responses, especially under salt stress. After salt stress treatment, the growth of the cultivated peanut Tifrunner is inhibited. Nine *NRT1* genes identified in Tifrunner, which respond to salt stress, help maintain the absorption and transport of nitrate in roots or leaves, enabling peanuts to adapt to salt stress conditions [14]. *GmNRT1.5A (Glycine max NRT1.5A)* is predominantly expressed in seeds and roots, and its expression in roots is significantly induced by salt and drought stress, as well as by ABA and ACC, while GA_3_ treatment significantly inhibits its expression [15]. Salt stress induces *MdNRT1.1 (Malus domestica NRT1.1)* expression, but overexpression of *MdNRT1.1* in *Arabidopsis* reduces salt tolerance, increases MDA content and relative conductivity, and leads to more ROS accumulation and severe cell damage, as shown by DAB and NBT staining [16]. In another interesting study, salt-tolerant *Populus euphratica* alleviates salt stress by upregulating NRTs to maintain NO_3_^−^ supply, while salt-sensitive *P. simonii* × (*P. pyramidalis* × *Salix matsudana*) (*P. popularis cv. 35–44*, *P. popularis*) shows reduced NO_3_^−^ uptake and limited nitrogen supply due to the decreased transcripts of NRT1.1, 2.4, and 3.1 after 7 days of salt stress [17]. These studies not only reveal the crucial role of nitrate transporters (NRTs) in regulating plant responses to salt stress but also provide new research insights into the adaptive mechanisms of salt-tolerant species and the potential limitations of salt-sensitive species.

*Suaeda glauca* is an annual herbaceous plant known for its strong stress resistance. It thrives in saline soil, lake shores, and coastal areas, exhibiting excellent salt-alkali tolerance by growing in conditions equivalent to 200 mM NaCl. Compared to other halophytes, *Suaeda glauca* has significant advantages, including its widespread natural distribution, high salinity tolerance, rich nutritional and medicinal components, and relatively small genome (approximately 500 MB), and the diploid *Suaeda glauca* has nine pairs of chromosomes [18,19,20,21]. This contrasts sharply with *Salicornia bigelovii*, whose genome size is 2026 Mb [22]. The small genome of *S. glauca* enhances its utility in salinity tolerance research, positioning it as an ideal model plant [18,23]. Saline-alkali soils severely degrade soil structure and fertility, significantly reducing crop yields [24,25]. *Suaeda glauca* mitigates these effects by absorbing soil salts, improving aeration, and lowering soil pH [18]. Its salinity tolerance and ecological adaptability make it a valuable resource for restoring soil fertility and ecological balance in saline-alkaline lands. Additionally, its genetic resources offer significant potential for breeding salt-tolerant crops, enhancing their adaptability and productivity in saline environments [18,23]. Transcriptomic data showed that the *Suaeda salsa NRT1.1D* gene (*SsNRT1.1D*) is significantly upregulated under salt stress. Transgenic tomatoes overexpressing *SsNRT1.1D* under the 35S promoter exhibited enhanced salt tolerance in field trials. This highlights the great potential of *Suaeda salsa NRT* genes in breeding crops for salt tolerance [26].

The development and utilization of saline-alkali lands pose a significant challenge to agricultural production. Nitrogen is crucial for plant growth, development, and salt tolerance [27]. Thus, *Suaeda glauca*, a typical halophyte, holds great potential for research and application of salt tolerance genes. However, comprehensive reports on the *NRT* gene family in halophytes are still limited. This study used *Arabidopsis thaliana* and some closely related species to *Suaeda glauca*, such as spinach (*Spinacia oleracea* L.), for reference to focus on the evolution and function of the *NRT* gene family in *Suaeda glauca*. This not only elucidates the mechanisms of nitrogen absorption and transport in plants but also offers new insights into improving plant nitrogen nutrition to enhance salt tolerance in plants.

## 2. Materials and Methods

### 2.1. Plant Samples and NaCl Concentration Treatments

In this study, *Suaeda glauca* seedlings (provided by Yancheng Lvyuan Salt Soil Agricultural Technology Co. Ltd., Yancheng, Jiangsu, China) were used as experimental material. After three weeks of growth on a nutrient substrate, seedlings with consistent growth were selected, cleaned, and the substrate was removed from their roots before transferring them to a Hoagland nutrient solution (Beijing Coolaber Technology Co., Ltd., Beijing, China) for further cultivation. To ensure a consistent nutrient supply, the nutrient solution was renewed every three days. The experimental group was subjected to high salt stress by adding 200 mM NaCl to the nutrient solutions. Samples were collected from 13 different organs or tissues (fibrous roots, taproot, main stem, lateral stem, main stem leaf, lateral stem leaf, apical meristem, calyx, petal, gynoecium, anther, ovary wall, stigma) and at various time points (0, 0.5, 2, 4, 8, 16, 24, and 48 h after salt treatment) for subsequent experimental analysis. Each treatment condition was replicated three times.

### 2.2. Identification of NRT Genes in Suaeda glauca

The genome assembly of *Suaeda glauca* has been published [23]. It has a size of 1.02 Gb (comprising two sets of haplotypes). Both raw data and genome assembly are available from the China National Center for Bioinformation (https://www.cncb.ac.cn/, accessed on 15 March 2025) under the accession numbers subCRA017428 and WGS038631. HMM profile files for the PTR2 (PF00854), MFS_1 (PF07690), and NAR2 (PF16974) domains were downloaded from the Pfam database (http://pfam.xfam.org/, accessed on 15 March 2025). The PTR2 domain is closely related to the proton-dependent transport of nitrogen-containing substrates and is a key feature for identifying members of the *NRT1* subfamily [28]. The MSF_1 domain is associated with ion transport and can be used to effectively identify NRT2 family members. NRT3 proteins typically contain an NAR2 domain, which plays an auxiliary role in nitrate transport, helping NRT2 proteins achieve efficient nitrate uptake and transport [29]. Potential [30] family members were identified based on these profiles using TBtools software (v2.315) [30]. Subsequently, the predicted gene sequences were verified for the presence of these domains using the Pfam website. Genes with missing or incomplete domains were then eliminated. Additionally, the protein sequences of the NRT family from *Arabidopsis thaliana* were obtained from the TAIR website (http://www.arabidopsis.org, accessed on 15 March 2025), and similar sequences were identified in the *Suaeda glauca* protein sequences using the Protein BLAST method to confirm the members of the gene family. All protein sequences obtained by these two methods were identified as members of *Suaeda glauca* NRT family after examination screening and will be used for subsequent research and analysis. The isoelectric point (pl.), molecular weight (MW), and the number of amino acids of the *Suaeda glauca* NRT proteins were predicted using TBtools software (v2.315). It should be noted that, owing to the large gene set, each candidate SgNRT was initially assigned a sequential genomic ID in ascending order, after which the corresponding family prefix—NRT1/NPF, NRT2 or NRT3—was appended to generate the final nomenclature.

### 2.3. Phylogenetic Analysis of NRT Families

All sequences used in this study are listed in the Supplementary file. The protein sequences of the *Suaeda glauca* and *Arabidopsis thaliana NRT* genes were compared and aligned using the MUSCLE tool in MEGA11.0 (Max Memory in MB = 2048, other parameters set to default). Phylogenetic trees were constructed using MEGA 11.0 with the neighbor-joining (NJ) method. The NJ parameters were as follows: Bootstrap method *n* = 1000, pairwise deletion of gaps, p-distance, and other parameters set to default. Finally, the phylogenetic tree was visualized using iTOL (https://itol.embl.de/, accessed on 20 March 2025). The sequences and classification nomenclature for SoNPF were based on a published study [31].

### 2.4. Motif, Conserved Domain, and Gene Structure Analysis of SgNRTs

To further characterize the gene structure, we used MEME (https://meme-suite.org/, accessed on 15 March 2025) to identify conserved nucleotide motifs (with the maximum number of motifs set to 15) using default parameters. The data were obtained through a batch CD search on the NCBI. Gene information was extracted from the gene structure annotation file to visualize the UTR and CDS lengths.

### 2.5. Chromosomal Localization and Synteny Correlation of NRT Family

To observe tandem and segmental duplications of the *Suaeda glauca NRT* genes, collinearity analysis was performed using MCScanX with the gene annotation file and the genome sequence of *Suaeda glauca*. The circos plot was constructed using Tbtools software (v2.315).

### 2.6. Cis-Element Analysis of SgNRT Gene Promoters

To identify cis-acting regulatory elements within the promoter regions, the sequences of *SgNRT*, consisting of 2000 nucleotides upstream, were extracted from the genome file. PlantCare (https://bioinformatics.psb.ugent.be/webtools/plantcare/html/, accessed on 15 March 2025) was used to detect cis-acting elements in the promoters.

### 2.7. Gene Expression Profiling of the NRT Genes

Transcriptome analysis was performed using transcriptome data provided by the Center for Genomics of Haixia Institute of Science and Technology of Fujian Agriculture and Forestry University. Expression data for the *SgNRT* gene family (FPKM values) were obtained from 13 different tissues, as well as the dynamic expression levels of *SgNRT* genes in roots and leaves under salt treatment from 0 to 48 h after treatment. Using TBTools, a heatmap of *SgNRT* gene expression across different tissues was generated.

## 3. Result

### 3.1. Genome-Wide Identification and Characterization of NRT Members in Suaeda glauca

To better understand the *NRT* gene family in *Suaeda glauca*, we used TBtools software (v2.315) to screen its protein sequences for specific domains. The protein sequences of the *NRT family* of *Arabidopsis thaliana* and *Suaeda glauca* were aligned for validation, and candidate NRT family members were identified. Given the large number, candidate SgNRT members were first ordered by ascending genomic ID, and then prefixed with their respective family (NRT1/NPF, NRT2, or NRT3) to yield the final designations (Supplementary Table S1). The predicted amino acid sequence lengths of the *SgNRT* gene family in *Suaeda glauca* vary roughly between 400 and 600.

Six SgNRT proteins (SgNRT46, SgNRT173, SgNRT134, SgNRT169, and SgNRT208) contained a large number of amino acids. The *NRT* gene family belongs to the MFS superfamily. In early studies, most members of the MFS superfamily consisted of 400–600 amino acid residues, and secondary structure predictions indicated that they predominantly possess 12 transmembrane α-helical domains. To further confirm these candidate members, TMHMM (https://services.healthtech.dtu.dk/services/TMHMM-2.0/, accessed on 15 March 2025) transmembrane helix (TMS) analysis and SWISS-MODEL (https://swissmodel.expasy.org/, accessed on 15 March 2025) 3D structure prediction were conducted. Results revealed that these six proteins have more than 20 transmembrane domains, likely evolved from the original 12 transmembrane α-helical domains [32]. Combined with the 3D structure prediction outcomes, the models show that these proteins exhibit the conformation of transport proteins on the biological membrane, while SgNRT12 appears to have two domains. Based on the structural domains and 3D protein models, these six SgNRT candidate proteins were not included in the subsequent analysis of the *SgNRT* gene family.

The only member identified in the SgNRT3 subfamily was SgNRT3.206, which comprised 735 amino acids. Additionally, the molecular weights of SgNRTs range from 38.86 kDa (SgNRT2.182) to 238.20 kDa (SgNRT2.38). The theoretical isoelectric points span from 4.90 (SgNRT2.18) to 9.82 (SgNRT2.157). The SgNRT members were divided into three families based on phylogenetic analysis, conserved domain analysis, and classification methods of *Arabidopsis thaliana*. Specifically, 61 genes were classified as SgNRT1/SgNPF, 150 as SgNRT2, and one as SgNRT3.

### 3.2. Evolutionary Relationships of NRT Members Between S. glauca and Other Species

**Figure 1:**
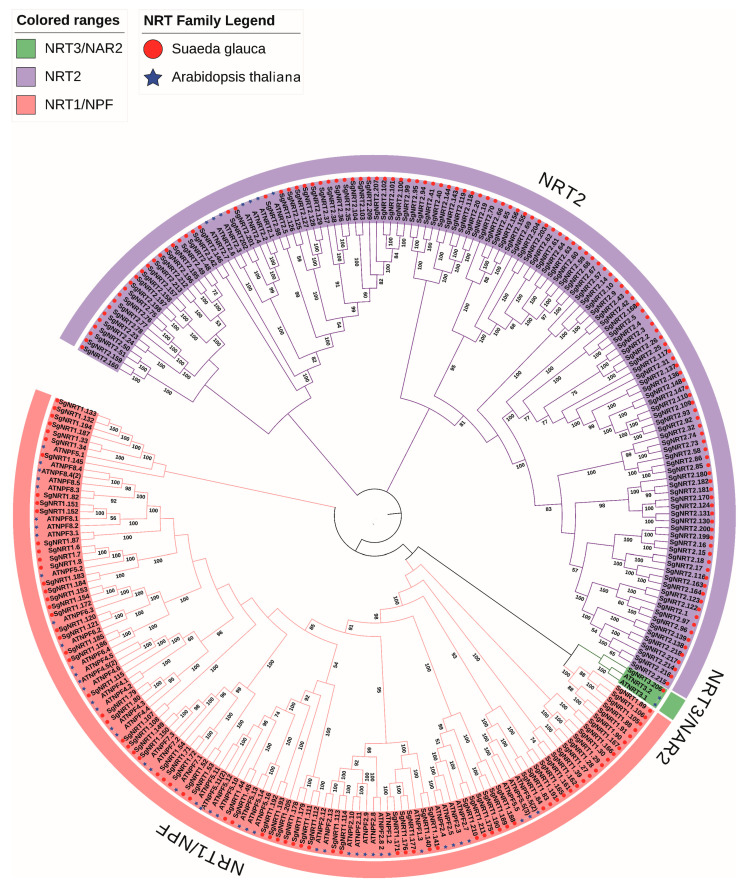
Phylogenetic tree of NRT protein from *Arabidopsis thaliana* and *Suaeda glauca*. A neighbor-joining phylogenetic tree was constructed using Molecular Evolutionary Genetics Analysis software (MEGA 11.0) based on a total of 266 protein sequences, including 54 from *Arabidopsis thaliana* and 212 from *Suaeda glauca*, with 1000 bootstrap replicates. A total of 61 members were identified as SgNRT1, 150 as SgNRT2, and one as SgNRT3. Members of the NRT family from *Arabidopsis thaliana* and *Suaeda glauca* are distinguished by blue stars and red circles, respectively.

To clarify the functional and evolutionary relationships of the *NRT* gene family, 54 AtNRT proteins from *Arabidopsis* and 212 SgNRT proteins from *Suaeda glauca* were used to construct a phylogenetic tree using the neighbor-joining method. *Arabidopsis thaliana*, a commonly used model plant in research, has well-studied gene functions. The results showed that all NRT proteins were divided into three subfamilies: NRT1/NPF, NRT2, and NRT3/NAR2. The SgNRT2 family, with 150 members, is the largest branch; the SgNRT1/SgNPF family has 61 members, and the SgNRT3 subfamily has only one member (Figure 1). All sequences used in this study are listed in the Supplementary file.

The SgNRT2 family, with its large number of members, likely plays a complex and diverse role in *Suaeda glauca* adaptation to the environment, participating in nitrate absorption and transport across different growth stages and under stress conditions. In contrast, the smaller SgNRT1 family may perform unique and critical functions, such as efficient nitrate uptake under low-nitrate conditions. The single member of the SgNRT3 family suggests highly specialized functions, possibly in long-distance nitrate transport. The rapid evolution and expansion of the SgNRT2 subfamily may be driven by *Suaeda glauca* adaptation to saline-alkali soils, generating functionally diverse members that meet nitrate demands under various conditions. This gene family expansion, compared to that in *Arabidopsis*, reflects the adaptive evolution of *Suaeda glauca* to cope with specialized environments. Additionally, certain members of the SgNRT2 family (from SgNRT2.214 to SgNRT2.169, clockwise) appeared to lack strong homology with *Arabidopsis thaliana*. Considering that *Arabidopsis* belongs to *Brassicaceae*, while *Suaeda glauca* belongs to *Chenopodiacea*, the phylogenetic distance between the two species is considerable. However, there is a scarcity of well-documented NRT gene families in halophyte plants with strong reference values. Therefore, the sequence similarity between the SgNRT2 subfamily members and AtNRT2 protein sequences from *Arabidopsis* was used as reference data for subsequent research (Supplementary Table S2). Phylogenetic tree of Suaeda glauca NRT2 and NRT2 members from other species. A total of 26 sequences from seven species, including *Ginkgo biloba*, *Populus trichocarpa*, *Pinus pinaster*, *Suaeda altissima*, *Chenopodium quinoa*, *Beta vulgaris*, and *Spinacia oleracea*, were selected to construct a phylogenetic tree with SgNRT2s. For the NRT2 family in *Suaeda glauca*, limited by the insufficient research results on the identification of the NRT2 family in other halophytes, the phylogenetic tree of NRT2 with other species did not achieve satisfactory visualization results (Figure S1).

**Figure 2:**
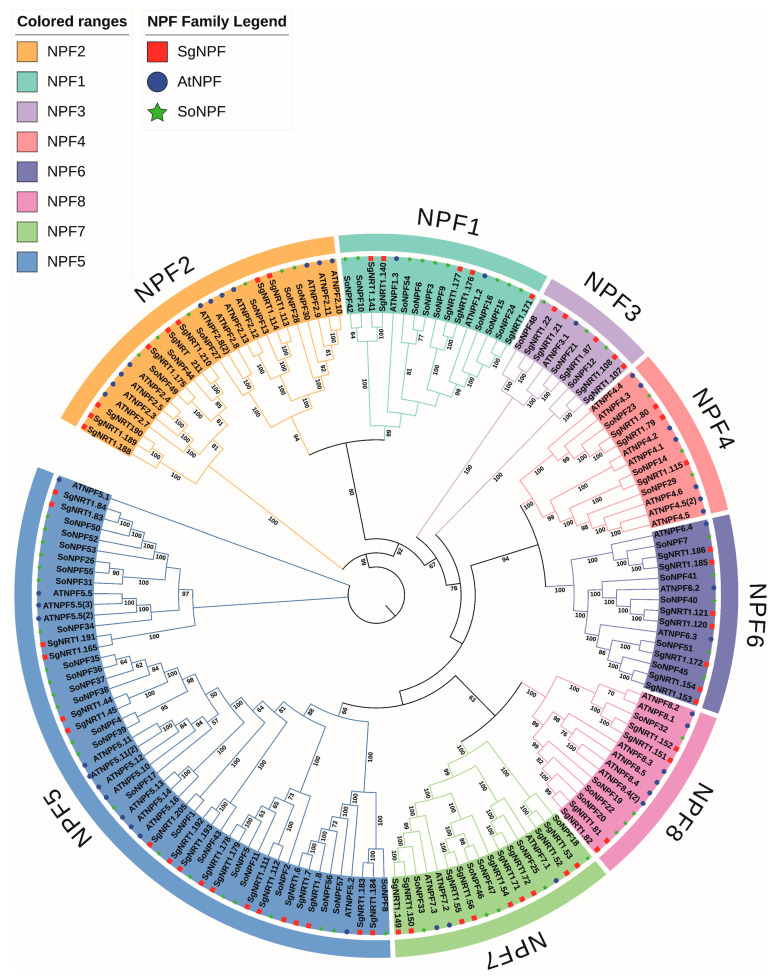
Phylogenetic tree of NPF subfamily proteins from *Arabidopsis thaliana*, spinach (*Spinacia oleracea* L.), and *Suaeda glauca*. The neighbor-joining phylogenetic tree was constructed using Molecular Evolutionary Genetics Analysis software (MEGA 11.0) based on 172 protein sequences, including 54 from *Arabidopsis thaliana*, 57 from spinach, and 61 from *Suaeda glauca*, with 1000 bootstrap replicates. Bootstrap values are indicated in the figure. A total of 5 members were identified as SgNPF1, 8 as SgNPF2, 4 as SgNPF3, 3 as SgNPF4, 18 as SgNPF5, 7 as SgNPF6, 9 as SgNPF7, and 4 as SgNPF8 members.

To facilitate a clearer classification of the NPF subfamilies, a phylogenetic tree was constructed using NPF members from spinach (*Spinacia oleracea* L.), which is more closely related to *Suaeda glauca*, and *Arabidopsis thaliana*, a well-characterized model plant (Figure 2, Table 1). The construction of the phylogenetic tree with SgNP family members also referenced the sequences and naming conventions of *Arabidopsis* and spinach. The sequences and classification nomenclature for SoNPF were based on published studies [31].

Table 1 Characteristics of *SgNPF* family members. SgNPFs can be further subdivided into eight groups. Based on the evolutionary relationships of the NPF subfamily members of the reference species, as well as the classification and naming of AtNPF and SoNPF members, the 61 SgNPF subfamily members were assigned SgNPF subfamily designations. The 61 individual SgNRT1 subfamily members retain their names within the SgNRT family and are also given detailed classification names within the SgNPF subfamily. The 61 SgNRT1 members were identified as follows: five as SgNPF1, eight as SgNPF2, four as SgNPF3, three as SgNPF4, 18 as SgNPF5, seven as SgNPF6, nine as SgNPF7, and four as SgNPF8.

**Figure 3:**
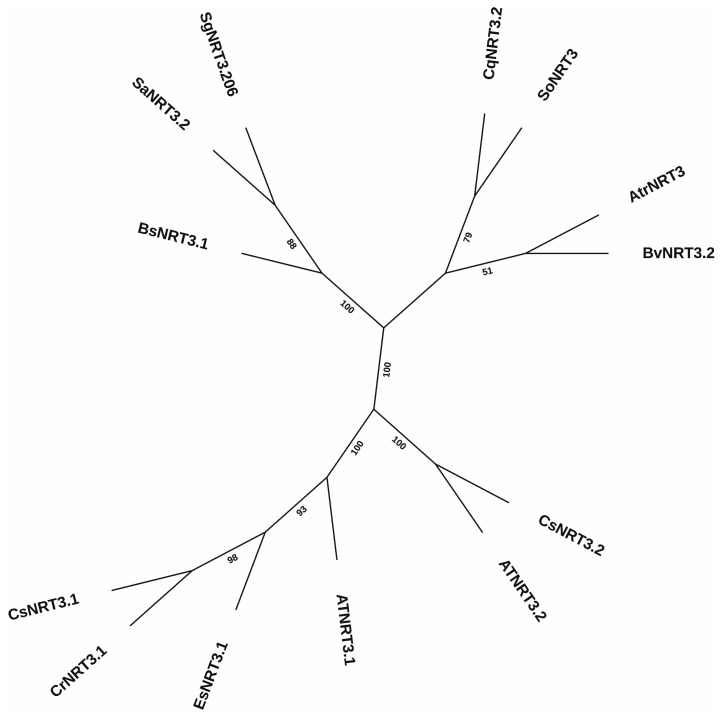
Phylogenetic tree of the NRT3 family protein. Protein sequences from Suaeda glauca, Arabidopsis thaliana, Spinach (*Spinacia oleracea* L.), *Chenopodium quinoa*, *Beta vulgaris*, *Amaranthus tricolor*, *Suaeda altissima*, *Bienertia sinuspersici*, *Camelina sativa*, *Eutrema salsugineum*, *Capsella rubella*, *Camelina sativa*. A neighbor-joining phylogenetic tree was constructed using Molecular Evolutionary Genetics Analysis software (MEGA 11.0) based on 13 protein sequences.

A phylogenetic tree was constructed using 13 NRT3 protein sequences from multiple species (Figure 3), including *Suaeda glauca*, *Arabidopsis thaliana*, *Spinacia oleracea* L. (spinach), *Chenopodium quinoa*, *Beta vulgaris*, *Amaranthus tricolor*, *Suaeda altissima*, *Bienertia sinuspersici*, *Camelina sativa*, *Eutrema salsugineum*, and *Capsella rubella*. In the phylogenetic tree, the NRT3 members from the two *Suaeda* species are the closest to each other. Next, other halophytes or salt-tolerant plants (such as *Chenopodium quinoa*, *Spinacia oleracea*, and *Bienertia sinuspersici*) are relatively close to *Suaeda glauca* NRT3. Conversely, the NRT3 members of *Suaeda glauca* were more distantly related to *Arabidopsis thaliana* and those with higher homology to AtNRT3.1 and AtNRT3.2 in the phylogenetic tree.

### 3.3. Motif, Conserved Domain, and Gene Structure Distribution of SgNRT

**Figure 4:**
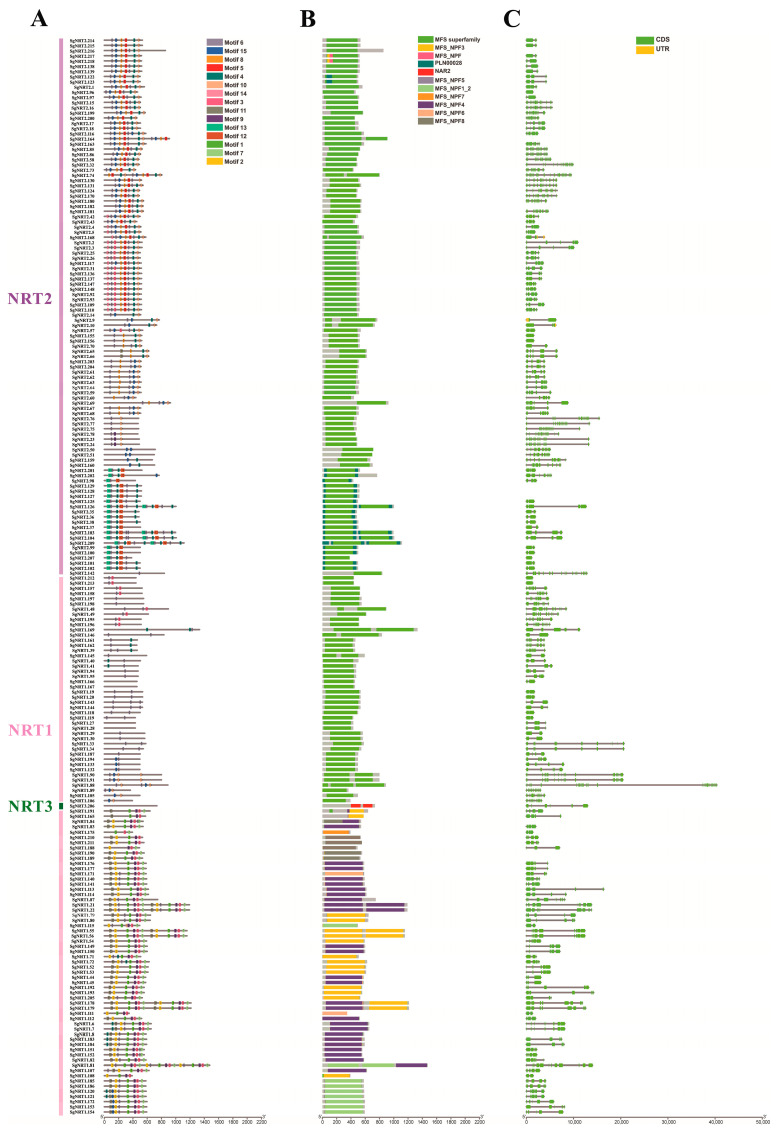
Motifs, conserved domains, and gene structures of SgNRTs. (**A**) Motif analysis of NRT protein sequences in *Suaeda glauca*. (**B**) Conserved domain analysis using the NCBI Batch CD-search tool. (**C**) Gene structure analysis. Yellow regions represent non-translated regions, the green regions represent coding sequences, and the uncolored lines represent introns.

To gain a deeper understanding of the structural characteristics and functional diversity of the *Suaeda glauca* NRT gene family, we analyzed the motifs, conserved domains, and gene structures of the identified SgNRT members. (Figure 4). However, due to the lack of information in the GFF genome annotation file, 15 genes were not detected in terms of gene structure, and all structures lack untranslated region (UTR) information (Figure 4C).

The conservation of NRT gene family members can be reflected by different features, such as conserved motifs, gene structure, and conserved domains. The motifs were arranged in a stable order on the NRT2 protein sequences (motifs 6, 15, 8, 6, 4, and 10). In NRT1, the motifs are arranged in the following order: motif 11, motif 2, motif 6, motif 1, motif 9, motif 3, and motif 7. No similar motifs were detected in the NRT3 member SgNRT206. Some members do not fully follow this order, which may be due to multiple expansion events in the NRT gene family. Motif 6 appeared in both NRT1 and some NRT2 members, indicating its potential role in nitrate or other substrate transport (Figure 4A).

Further validation was performed through a comprehensive analysis of diverse superfamily conserved domains, including MFS-NPF1-2, MFS-NPF3, MFS-NPF4, MFS-NPF5, MFS-NPF6, MFS-NPF7, MFS-NPF8, MFS-superfamily, PLN00028, and NAR2 domains (Figure 4B) [33,34]. Notably, some members possess both MFS and PLN00028 conserved domains, suggesting gene duplication and expansion events, and their specific functions remain to be investigated.

Among the 15 motifs (Figure S2), higher conservation is indicated by taller amino acid symbols, indicating stable sequence patterns that often correlate with biological functions. For example, the high conservation of Motif 14 underscores its functional importance. Conversely, the relatively low conservation of Motif 4, indicated by shorter symbols, suggests variability and potential evolutionary changes in SgNRT1 and SgNRT2 members, possibly linked to gene family expansion or sequence disorder.

### 3.4. Amplification and Evolutionary Patterns of SgNRT Genes

**Figure 5:**
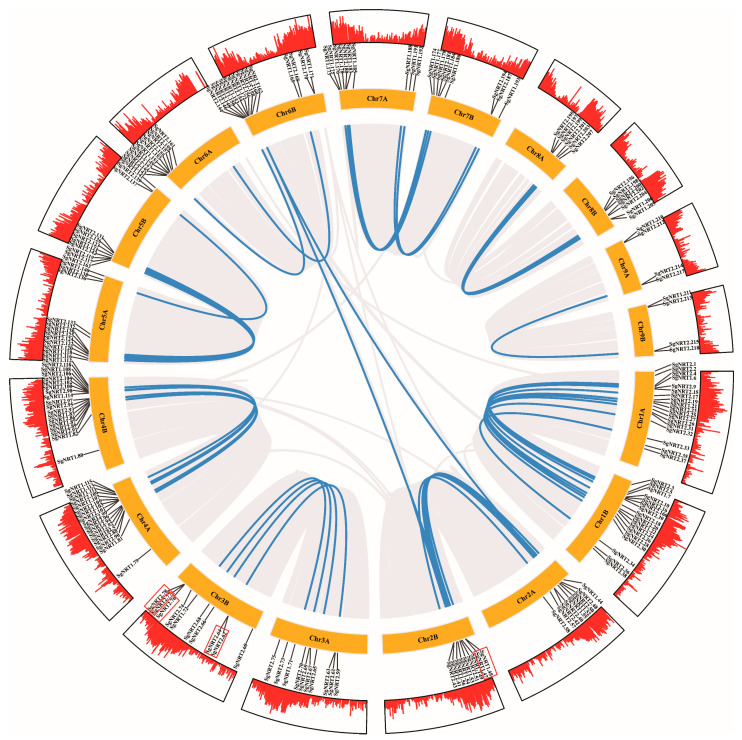
Collinearity relationships of SgNRTs in *Suaeda glauca*. The chromosomal localization and collinearity of SgNRT genes are shown. Gray lines denote collinear gene pairs within the genome, and blue lines highlight collinear pairs within the SgNRT family. The middle circle shows the chromosomal localization of SgNRT genes, and the red frames mark tandem duplication events. The outer circle illustrates gene density across chromosomes using bar plots.

To reveal the evolutionary characteristics of *SgNRT* gene families and to display collinearity relationships and gene duplication events in the *Suaeda glauca* genome, a Circos plot was constructed using TBtools (Figure 5). Blue lines clearly depict the relationships between collinear gene pairs, revealing the distribution and arrangement of *SgNRT* genes across chromosomes. These lines also indicate potential duplication and rearrangement events in the *SgNRT* gene family during evolution, implying that these gene pairs may have maintained similar functions and regulatory mechanisms.

Chromosomes such as Chr1A, Chr1B, Chr2A, Chr2B, Chr4A, Chr4B, Chr5A, Chr5B, Chr7A, and Chr7B contained a higher number of collinear gene pairs. This may indicate that these regions have been relatively stable during evolution or have undergone frequent gene duplication and recombination events. Additionally, collinear lines between different chromosomes, such as Chr2A, Chr2B, and Chr6B, suggest possible chromosomal rearrangements or translocation events that may have occurred during the evolution of the species.

A total of 6 tandem-duplicated pairs (14 genes) were identified, including *SgNRT2.9*, *SgNRT2.10*, *SgNRT2.12*, *SgNRT1.44*, *SgNRT1.45*, *SgNRT1.47*, *SgNRT2.62*, *SgNRT2.64*, *SgNRT2.76*, *SgNRT2.77*, *SgNRT2.78*, *SgNRT2.214*, and *SgNRT2.217*. Red frames highlight seven of these pairs. Notably, *SgNRT217* was not annotated due to missing information in the annotation file. These pairs were mainly located in the chromosomal regions of Chr1A, Chr1B, Chr2A, Chr2B, and Chr3B, indicating higher activity of gene duplication and evolution in these regions. Genes resulting from tandem repeat events may have the same molecular function but participate in different biological processes. The presence of these tandem-duplicated gene pairs reveals dynamic changes that the gene family may have undergone during evolution, providing important clues for studying the functional diversification and adaptive evolution of the SgNRT families.

### 3.5. Characterization of Cis-Acting in the SgNRT Genes Promoter Region

**Figure 6:**
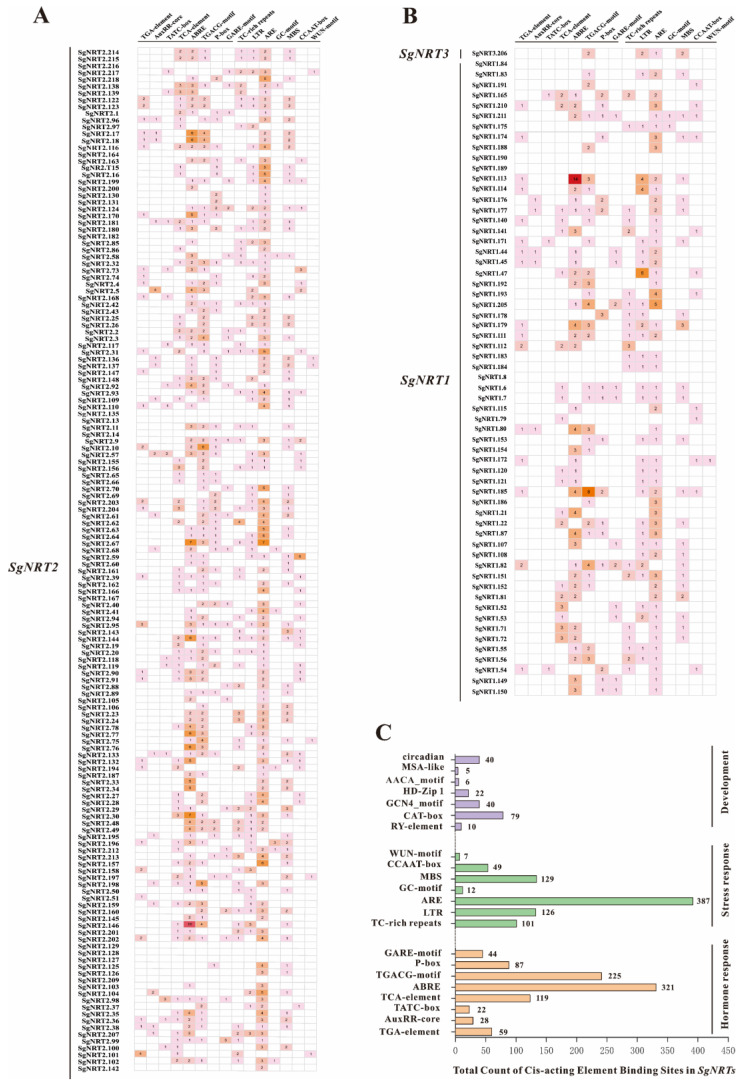
Cis-acting regulatory elements (CREs) in the promoter regions of *SgNRT* genes. (**A**) Distribution and abundance of hormone- and stress-responsive CREs in the promoter regions of *SgNRT2* family members. (**B**) Distribution and abundance of hormone-responsive and stress-responsive CREs in the promoter regions of *SgNRT3* and *SgNRT1* family members. (**C**) The Y-axis shows 22 CREs across three categories, and the X-axis indicates the total number of CRE-binding sites in *SgNRT* gene promoter regions.

To elucidate the regulatory mechanisms of *SgNRT* genes under various environmental conditions, cis-acting element (CREs) analysis was conducted to identify key regulatory sequences in the promoter regions that may be involved in the activation or repression of gene expression. Functional cis-acting components related to hormone regulation and stress response are illustrated by a heat map (Figure 6A,B). Notably, the promoter regions of two members, SgNRT1.113 and SgNRT2.146, contained a high number of abscisic acid response element (ABRE) cis-acting element binding sites, indicating that these genes may be highly responsive to abscisic acid (ABA). Specifically, the promoter of SgNRT1.113 has 14 binding sites, while that of SgNRT2.146 has 16. ABA is a crucial plant hormone involved in stress responses, including drought and salt stress responses. Thus, SgNRT1.113 and SgNRT2.146 likely contribute to stress tolerance mechanisms, aiding plant survival under adverse conditions.

In addition to being associated with hormones and stress responses, *NRT* genes are also related to development (Figure 6C). Specifically, the promoter regions of *SgNRT* genes contained 321 abscisic acid-responsive ABRE-binding sites, 225 methyl jasmonate (MeJA) responsive TGACG motifs, and 119 salicylic acid-associated TCA elements. Auxin signaling was indicated by 28 AuxRR-core and 59 TGA-elements, while gibberellin responsiveness was marked by 44 GARE-motifs, 22 TATC-boxes, and 87 P-boxes. Stress-responsive CREs included 387 AREs, 129 MBS motifs linked to anaerobic and drought responses, 126LTRs, 101 TC-rich repeats, and 7 WUN-motifs associated with cold, defense, and wounding. Developmental elements featured 40 circadian regulators, 79 CAT-boxes for meristem activity, 22 HD-Zip 1 elements associated with differentiation, 6 AACA motifs involved in endosperm-specific negative expression, 5 MSA-like motifs, and 10 RY-elements for seed maturation. These findings suggest that *SgNRT* genes are regulated by diverse hormonal and stress signals and play multifunctional roles in plant development and adaptation.

### 3.6. The Specific Expression of SgNRT Genes in 13 Different Organs or Tissues

**Figure 7:**
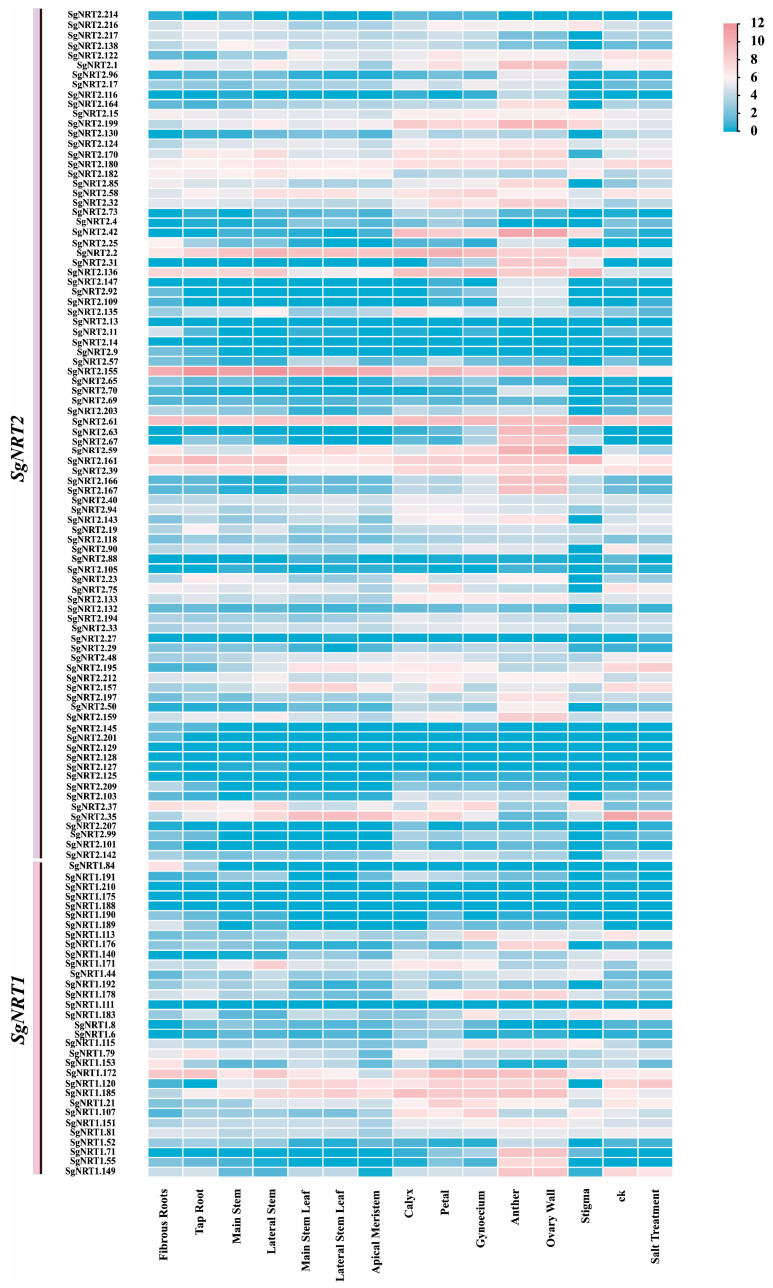
Specific expression of *SgNRT* genes in 13 different organs or tissues. The heatmap represents the expression of *SgNRT* genes in fibrous roots, taproot, main stem, lateral stem, main stem leaf, lateral stem leaf, apical meristem, calyx, petal, gynoecium, anther, ovary wall, stigma, ck, and salt (200 mM) treatment. The ck and salt treatment groups used *Suaeda glauca* leaves.

To further understand the function of *SgNRT* genes, transcriptome data analysis revealed that *SgNRT* genes exhibit distinct tissue-specific expression patterns across 13 different tissues in *Suaeda glauca* (Figure 7). Unfortunately, transcriptome data for the *SgNRT3* family are lacking. Notably, *SgNRT2.42*, *SgNRT2.31*, *SgNRT2.63*, *SgNRT2.67*, *SgNRT2.166*, *SgNRT2.167*, *SgNRT2.113*, *SgNRT1.176*, and *SgNRT1.71* show relatively high expression levels in reproductive organs, which may suggest their roles in organ development and functions, including gamete formation, fertilization, and seed development. Most genes expressed in the roots and stems were also expressed in other organs or tissues. Except for *SgNRT1.84*, which was only expressed in fibrous roots and not in other tissues, several genes showed relatively higher expression in roots and stems than in other tissues. These include *SgNRT2.2*, *SgNRT2.155*, *SgNRT2.61*, *SgNRT2.161*, and *SgNRT1.172*. In contrast, *SgNRT2.2*, *SgNRT2.155*, *SgNRT2.35*, *SgNRT1.120*, and *SgNRT1.185* have relatively higher expression in leaves compared to other genes. These findings provide insights into the potential sites of action of NRT in *Suaeda glauca*.

Additionally, eight genes responded to salt stress, with their expression either increasing or decreasing after salt treatment. *SgNRT2.61* and *SgNRT1.171* show increased expression after salt treatment, while *SgNRT2.2*, *SgNRT2.155*, *SgNRT2.61*, *SgNRT2.35*, *SgNRT1.115*, *SgNRT1.153*, and *SgNRT1.149* are downregulated. The upregulation of *SgNRT2.61* expression after salt treatment may reflect an adaptive response to salt stress. Highly expressed *SgNRT2.61* likely participates in nitrogen metabolic processes under salt stress to facilitate plant adaptation. Although the expression of *SgNRT2.155* decreased after salt treatment, its high basal expression across organs suggests that it maintains essential biological functions under normal conditions, potentially adjusting its expression to sustain salt stress survival. Notably, *SgNRT2.35*, despite downregulation, retained relatively higher expression levels than other genes, hinting at an adaptive strategy to maintain high expression for necessary nitrogen nutrition under salt stress. These results preliminarily demonstrate that *SgNRT* genes respond to salt stress, but the specific response patterns need to be further verified.

### 3.7. The Dynamic Expression of SgNRT Genes Within 48 h After Salt Treatment

To further explore the temporal expression of *SgNRT* genes under salt stress, a detailed transcriptome analysis was conducted on leaf and root tissues from 0 to 48 h post-treatment. This analysis helped elucidate the dynamic expression patterns and regulatory mechanisms of *SgNRT* genes in the initial response and adaptation of plants to salt stress. The expression of *SgNRT* genes in leaves and roots shows dynamic changes. Most genes respond weakly to salt stress, while those that respond seem to display complex patterns. This suggests unique signaling or regulatory mechanisms for *SgNRT* genes under salt stress.

**Figure 8:**
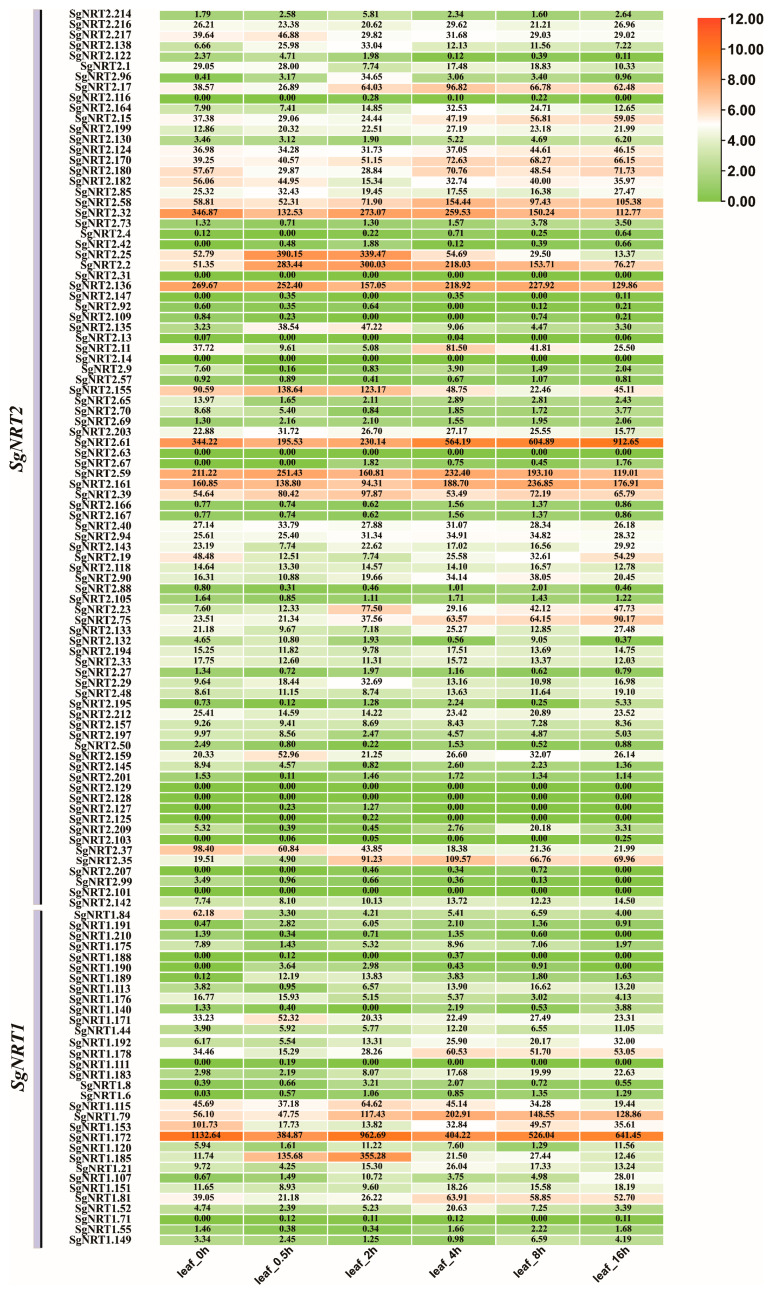
Expression characteristics of *SgNRT* genes within 48 h of salt treatment in leaves. Transcriptome data from leaf tissues at 0, 0.5, 2, 4, 8, 16, 24, and 48 h after salt treatment were analyzed. A logarithmic transformation was applied to the raw data to generate a heatmap.

In leaves, *SgNRT2.25*, *SgNRT2.2*, *SgNRT2.35*, and *SgNRT1.185* expression quickly increased within 0.5 h of salt treatment, peaking at 2 h, with levels appearing higher than those of the other genes (Figure 8). *SgNRT1.185* expression increased 32-fold within 2 h, while *SgNRT2.25*, *SgNRT2.2,* and *SgNRT2.35* expression increased 7.5-fold, 5.8-fold, and 5.7-fold, respectively. *SgNRT2.25*, *SgNRT2.35*, and *SgNRT2.2* all belong to the *SgNRT2.2* gene family, while *SgNRT1.185* belongs to the *SgNRT2.1* gene family. In contrast, *SgNRT2.32* initially decreased, then gradually increased, and stabilized at higher levels within 2 h. *SgNRT2.61* decreased initially, then increased to a peak at 16 h before returning to baseline. *SgNRT1.172* had the highest basal expression, which briefly dropped before recovering and maintaining a high expression level. Among these genes, *SgNRT2.25*, *SgNRT2.2*, *SgNRT2.32*, and *SgNRT2.61* belong to the *SgNRT2.2* family. *SgNRT1.185* and *SgNRT1.172* are members of the *SgNRT1* family.

Interestingly, *SgNRT2.35* and *SgNRT1.185* were induced by high salt concentrations (200 mM) in both leaves and roots (Figure 8). *SgNRT2.35* was only 2-fold upregulated, peaking at 8 h and maintaining high expression, with both basal and stress-induced levels higher than those of the other genes. It sustained a relatively high expression for 24 h. *SgNRT1.185* also showed a similar response in the roots. *SgNRT1.185* initially increased its expression by 2-fold within 2 h, then experienced a slight decrease in expression from 2 to 4 h, but by 8 h, it showed a 16-fold upregulation compared to the control (ck). Additionally, *SgNRT1.120* was upregulated early (within 2 h), slightly decreased, peaked again at 16 h, and returned to baseline. These findings indicate that analyzing temporal expression patterns of *SgNRT* genes after salt treatment can reveal their functions and regulatory mechanisms in plant salt stress responses. This includes identifying early response genes involved in rapid perception and signal transduction, as well as late-response genes associated with long-term adaptation. *SgNRT2.35* belongs to the *SgNRT2.2* family, while *SgNRT1.185* and *SgNRT1.120* are part of the *SgNRT1* family.

## 4. Discussion

Nitrates serve as the primary nitrogen source for higher plants and are linked to enhanced stress resistance through improved nitrogen utilization [12]. Plants typically acquire nitrates from soil via nitrate transport proteins [35]. Currently, the NRT gene family has been widely identified in Pecan [36], *Ginkgo biloba* [34], *Coffea* [37], soybean [38], barley [39], and other species. In *Arabidopsis*, 62 *NRT* gene family members have been identified, with *NRT1* having 53 members, the largest subfamily, while *NRT2* and *NRT3* families contain only seven and two family members [12]. Nevertheless, few studies have reported on the *Suaede glauca NRT* gene family. In this study, *NRT* gene family members were identified in the salt-tolerant plant *Suaeda glauca*, including 212 members that can be divided into three categories, with 61, 150, and 1 subfamily members, respectively (Figure 1). Phylogenetic analysis indicated rapid evolution within the NRT2 family, which is characterized by extensive branching. A total of 76 *PkNRT* genes (66 *NRT1/NPFs*, 6 *NRT2s*, and 4 *NRT3*) have been identified in Korean pine [40], 57 *VvNRT* genes (53 *NRT1/NPFs*, 3 *NRT2s*, and 1 *NRT3*) have been identified in grapes [41], and 84 NRT genes (66 *NRT1/NPFs*, 6 *NRT2s*, and 4 *NRT3*) have been identified in apples [27]. In the NRT1 family, the most classical study is that of *NRT1.1*. In *Arabidopsis thaliana*, there is only one *AtNRT1.1*, whereas in rice, three homologous genes have been identified: *OsNRT1.1A*, *OsNRT1.1B*, and *OsNRT1.1C* [42]. *OsNRT1.1B*, which is localized to the plasma membrane, is a functional homolog of *AtNRT1.1* and mediates nitrate signaling. It transmits nitrate signals to downstream nitrate responses via calcium-dependent and calcium-independent pathways. In simple terms, it may be responsible for sensing nitrogen in the environment, triggering downstream nitrate reactions. In contrast, *OsNRT1.1A*, which is localized in the vacuolar membrane, likely perceives the intracellular nitrogen status to fine-tune nitrogen metabolic processes.

In this study, we identified a greater number of NRT family members in *Suaeda glauca*, all of which contained similar structural domains. On the one hand, this is due to the genome assembly of *Suaeda glauca*, which includes two sets of haplotypes. On the other hand, the NRT family may have evolved and diversified through being involved in more processes in different environments under varying demands. Conversely, the NRT family may have evolved and diversified through increased functional demands. For instance, there are significant structural differences between *NRT2* genes in monocots and dicots. Phylogenetic reconstruction suggests that *NRT2* genes primarily evolved after the divergence of monocots and dicots, with distinct evolutionary patterns observed among *NRT2* genes in different plant species [43]. Similarly, differentiation within the NRT family exists among different subspecies. In rice, there are two subspecies: *japonica* and *indica.* The gene *OsNRT1.1B* diverged between *japonica* and *indica*. The 980th base of the *NRT1.1B* gene is a cytosine (C) in *japonica*, and a thymine (T) in *indica*, resulting in the 327th amino acid of the *NRT1.1B* protein being serine in *japonica* and isoleucine in *indica*. This SNP difference at the 980th position contributes to better nitrogen use efficiency (NUE) in *indica* rice [44].

In addition, the functional diversity of transporter protein families may be constrained by the arrangement and number of their structural domains, which also affects their potential for functional divergence during evolution. Channel proteins typically consist of polypeptide chains with 1 to 6 TMSs, whereas carrier proteins usually exhibit 10 to 14 TMSs per polypeptide chain. This reflects the possible variation in protein length that may have occurred during the evolutionary process of the NRT gene family [32,45]. Members of transporter families may specialize in transporting a single type of molecule or exhibit a broad substrate specificity. The Nitrate and Peptide Transporter (NPF) family demonstrates a wider range of substrate specificities, including the transport of various plant-specific metabolites, such as anti-nutritional compounds, flavonoids, and steroidal glycoalkaloids [46]. In this study, it remains unclear whether the expansion of the *SgNRT* gene family, particularly *SgNRT2*, is related to *long-term salt stress exposure* in *Suaeda glauca*, if similar evolutionary patterns exist in other halophytes, and how many of the expanded genes function as dominant genes. To address these questions, further experimental evidence and evolutionary data from closely related and ecologically similar species are required. Currently, there is limited research on *NRT* gene families in halophytes, and this study provides preliminary data on the evolutionary events of *NRT* gene families in halophytes.

Soil salinity is a significant issue that affects plant growth and development. This can lead to a reduction in crop yields of up to 58% [47]. *Suaeda glauca* is a true halophyte capable of growing normally under 200 mM NaCl and can become a dominant species in saline-alkali soils [48,49]. The study of expression levels of *SgNRT* genes across various organs and tissues indicated distinct organ or tissue specificity for *SgNRT* gene expression (Figure 7). The differential expression of SgNRT genes across various organs and tissues reveals their functional differentiation in different parts of *Suaeda*. Specifically, *SgNRT1.84* is exclusively expressed in roots, while *SgNRT2.2*, *SgNRT2.155*, *SgNRT2.61*, *SgNRT2.161*, and *SgNRT1.172* show higher expression in roots and stems. In contrast, *SgNRT2.2*, *SgNRT2.155*, *SgNRT2.35*, *SgNRT1.120*, and *SgNRT1.185* are highly expressed in leaves. These expression patterns indicate that *SgNRT* genes have organ-specific expression. Nitrate transporters expressed in the roots may participate in nutrient absorption in *Suaeda glauca* from the external environment, a process generally regulated by hormones [50].

**Figure 9:**
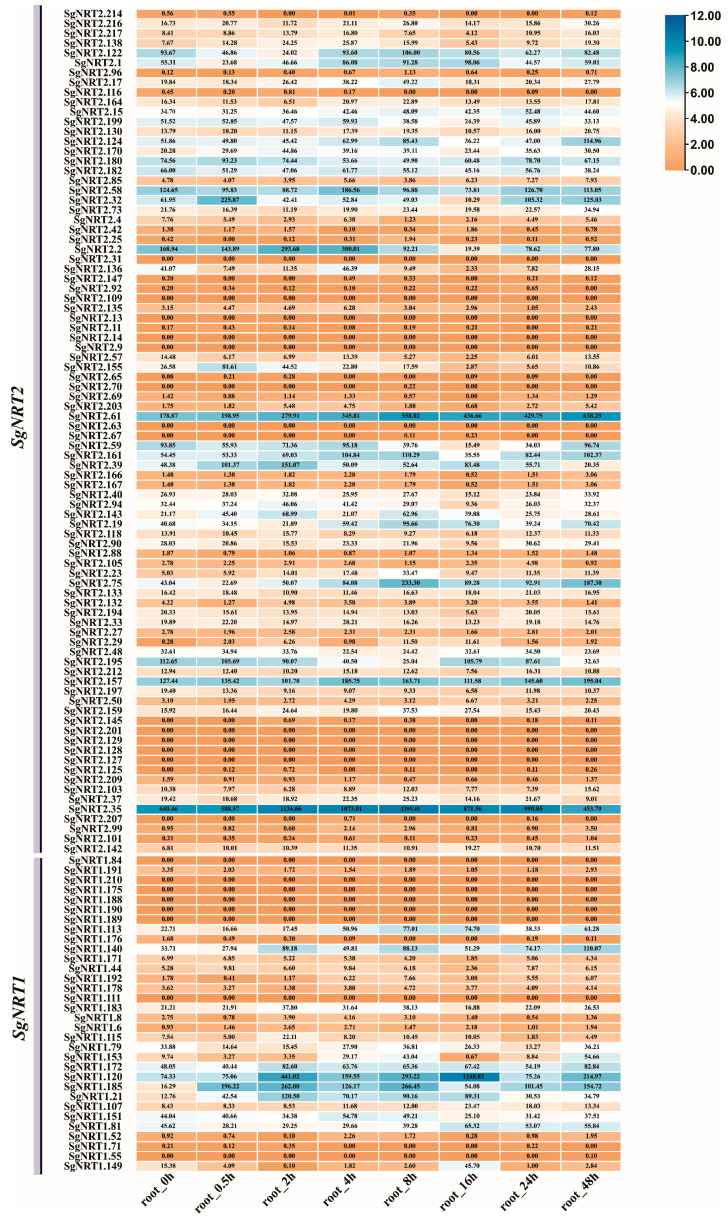
The expression characteristics of *SgNRT* genes within 48 h of salt treatment in roots. Transcriptome data from root tissues at 0, 0.5, 2, 4, 8, 16, 24, and 48 h after salt treatment were analyzed. A logarithmic transformation was applied to the raw data to generate a heatmap.

Many studies have found that salt stress may have varying impacts on nitrogen absorption and utilization efficiency in plants [51,52,53]. In response to salt stress, genes like *SgNRT2.25*, *SgNRT2.2*, and *SgNRT1.185* rapidly increased in expression within 0.5 h of salt treatment and peaked at 2 h, with expression levels higher than those of the other genes (Figure 8). These genes are likely involved in rapid perception and signal transduction, helping plants to quickly activate their defense mechanisms. In contrast, *SgNRT2.32*, *SgNRT2.35*, *SgNRT2.61*, *SgNRT1.172*, and *SgNRT1.120* show expression changes later in the stress response (Figure 8 and Figure 9), suggesting their involvement in long-term adaptation mechanisms of plants. The genes responding in roots suggest that they may be involved in the process of nitrogen nutrient absorption from the external environment under high salinity conditions (200 mM) in *Suaeda glauca*. Among them, *SgNRT2.35* had a higher basal level than the other genes without salt treatment and still showed induced upregulation after high salt induction, suggesting that it might be a dominant gene within the *SgNRT* gene family. However, this conclusion is currently only a possibility provided by transcriptome data and requires further experimental data for confirmation. In the phylogenetic analysis, *SgNRT2.35* corresponds to the homologous gene *AtNRT2.5* in *Arabidopsis thaliana*, while *SgNRT1.185* corresponds to *AtNPF6.4*. Previous studies have shown that *AtNRT2.5* is mainly expressed in the roots of Arabidopsis, especially under nitrate-deficient conditions, which is consistent with our findings [54]. Although there are reports on *AtNPF6.4*, it has been mainly reported to be expressed in the roots of rice and sugarcane, where it regulates tillering [55,56]. Therefore, whether *NPF6.4* has new functions in *Suaeda glauca* remains to be explored and verified in future research. To further validate the functions of these key genes in the future, transgenic overexpression in model plants (such as Arabidopsis thaliana) can be employed to assess whether they can significantly enhance the salt tolerance and nitrogen-use efficiency of the host plant. Additionally, using CRISPR technology to create multi-gene knockout lines could help elucidate their redundancy and necessity in the early response to salt stress. Although the regulatory network of these gene expressions remains to be further explored, it has already provided some potential candidate genes for the mining of nitrogen use efficiency genes in halophytes. In subsequent research, particular attention should be paid to whether genes that show differential expression under salt stress might serve as dominant genes for *Suaeda glauca* under salt stress conditions or be related to nitrogen-use efficiency.

However, some data gaps have impacted our analysis and conclusions. Firstly, structural information for 15 genes is missing. This restricts detailed functional domain and 3D structure analysis, adding uncertainty to the prediction of protein functions and interactions. Secondly, due to genomic annotation limitations, UTR information for all genes is unavailable. UTRs are crucial for the regulation of gene expression, including mRNA stability, translation efficiency, and subcellular localization. The absence of UTR data limits our understanding of post-transcriptional regulation and overall gene expression patterns. Thirdly, the lack of transcriptomic data for the SgNRT3 family constrains the analysis of its expression patterns. Without this data, we cannot assess how these genes respond to different conditions, particularly salt stress, and their potential roles in plant stress adaptation remain underexplored. In summary, these data gaps affect our understanding of the functions and regulatory mechanisms of the SgNRT gene family and may limit the comprehensiveness and accuracy of our conclusions. Future research should focus on obtaining missing data through improved genomic annotation and transcriptomic sequencing to better understand the role of the *SgNRT* gene family in plant salt tolerance.

Bioinformatics analysis revealed potential molecular targets for enhancing crop nitrogen-use efficiency and stress resistance. Future research should thoroughly explore the interactions among gene family members and the regulatory mechanisms of nitrate absorption and transport within plants to improve crop nitrogen-use efficiency and stress resistance.

## 5. Conclusions

Our findings suggest that the expression of *SgNRT* genes is spatially specific and may be involved in nitrogen absorption and transport to varying degrees in different developmental stages and organs of *Suaeda glauca*. *SgNRT1.185*, *SgNRT2.25*, and *SgNRT2.2* exhibit rapid salt-induction in leaves (activated within 0.5 h, peaking at 2 h), with *SgNRT1.185* showing a relatively high upregulation. *SgNRT1.185* and *SgNRT2.35* were induced by high salt concentrations (200 mM) in both roots and leaves. *SgNRT2.35* exhibited higher basal and stress-induced levels than the other genes. The preliminary investigation of salt stress-responsive genes aims to provide new genetic resources for future research.

## Figures and Tables

**Table 1:** Characteristics of SgNPF family members.

SgNPF Family	ID	SgNPF Family	ID
SgNPF1	SgNRT1.140/SgNPF1.1	SgNPF5	SgNRT1.165/SgNPF5.10
SgNPF1	SgNRT1.141/SgNPF1.2	SgNPF5	SgNRT1.178/SgNPF5.11
SgNPF1	SgNRT1.171/SgNPF1.3	SgNPF5	SgNRT1.179/SgNPF5.12
SgNPF1	SgNRT1.176/SgNPF1.4	SgNPF5	SgNRT1.183/SgNPF5.13
SgNPF1	SgNRT1.177/SgNPF1.5	SgNPF5	SgNRT1.184/SgNPF5.14
SgNPF2	SgNRT1.113/SgNPF2.1	SgNPF5	SgNRT1.191/SgNPF5.15
SgNPF2	SgNRT1.114/SgNPF2.2	SgNPF5	SgNRT1.192/SgNPF5.16
SgNPF2	SgNRT1.175/SgNPF2.3	SgNPF5	SgNRT1.193/SgNPF5.17
SgNPF2	SgNRT1.188/SgNPF2.4	SgNPF5	SgNRT1.205/SgNPF5.18
SgNPF2	SgNRT1.189/SgNPF2.5	SgNPF6	SgNRT1.120/SgNPF6.1
SgNPF2	SgNRT1.190/SgNPF2.6	SgNPF6	SgNRT1.121/SgNPF6.2
SgNPF2	SgNRT1.210/SgNPF2.7	SgNPF6	SgNRT1.153/SgNPF6.3
SgNPF2	SgNRT1.211/SgNPF2.8	SgNPF6	SgNRT1.154/SgNPF6.4
SgNPF3	SgNRT1.21/SgNPF3.1	SgNPF6	SgNRT1.172/SgNPF6.5
SgNPF3	SgNRT1.22/SgNPF3.2	SgNPF6	SgNRT1.185/SgNPF6.6
SgNPF3	SgNRT1.87/SgNPF3.3	SgNPF6	SgNRT1.186/SgNPF6.7
SgNPF3	SgNRT1.107/SgNPF3.4	SgNPF7	SgNRT1.52/SgNPF7.1
SgNPF3	SgNRT1.108/SgNPF3.5	SgNPF7	SgNRT1.53/SgNPF7.2
SgNPF4	SgNRT1.79/SgNPF4.1	SgNPF7	SgNRT1.54/SgNPF7.3
SgNPF4	SgNRT1.80/SgNPF4.2	SgNPF7	SgNRT1.55/SgNPF7.4
SgNPF4	SgNRT1.115/SgNPF4.3	SgNPF7	SgNRT1.56/SgNPF7.5
SgNPF5	SgNRT1.6/SgNPF5.1	SgNPF7	SgNRT1.71/SgNPF7.6
SgNPF5	SgNRT1.7/SgNPF5.2	SgNPF7	SgNRT1.72/SgNPF7.7
SgNPF5	SgNRT1.8/SgNPF5.3	SgNPF7	SgNRT1.149/SgNPF7.8
SgNPF5	SgNRT1.44/SgNPF5.4	SgNPF7	SgNRT1.150/SgNPF7.9
SgNPF5	SgNRT1.45/SgNPF5.5	SgNPF8	SgNRT1.81/SgNPF8.1
SgNPF5	SgNRT1.83/SgNPF5.6	SgNPF8	SgNRT1.82/SgNPF8.2
SgNPF5	SgNRT1.84/SgNPF5.7	SgNPF8	SgNRT1.141/SgNPF8.3
SgNPF5	SgNRT1.111/SgNPF5.8	SgNPF8	SgNRT1.152/SgNPF8.4
SgNPF5	SgNRT1.112/SgNPF5.9		

## Data Availability

Data are contained within the article or Supplementary Materials.

## Supplementary Materials

The following supporting information can be downloaded at: https://www.mdpi.com/article/10.3390/biology14081097/s1, Figure S1: The phylogenetic tree of Suaeda glauca NRT2 and NRT2 members from other species; Figure S2: Logos of 15 conserved motifs in *Suaeda glauca*; Supplementary File S1: Table S1: Characteristics of Candidate SgNRT and Associated Proteins; Table S2: The sequence similarity between the SgNRT2 subfamily members and AtNRT2 protein sequences; Table S3: The raw data of FPKM values of SgNRT genes in the 13 tissues; Table S4: The raw data of FPKM values of SgNRT genes within 48 hours in leaf; Table S5: The raw data of FPKM values of SgNRT genes within 48 hours in root; Supplementary File S2: All the protein sequences in *Suaeda glauca*, *Arabidopsis thaliana* and *Spinacia oleracea* L.

## Abbreviations

SgNRTs: *Suaeda glauca* Nitrate Transporter proteins; PTR2: Peptide Transporter 2; MFS_1: Major Facilitator Superfamily member; NAR2: Nitrate Assimilation Related 2; MEME: Multiple EM for Motif Elicitation; Blastp: Basic Local Alignment Search Tool for proteins; NCBI: National Center for Biotechnology Information; Batch CD-search: Batch Conserved Domain Search; TGA-element: Auxin-responsive element; AuxRR-core: Auxin Response Region core; TATC-box: Gibberellin-responsive element; TCA-element: Salicylic acid-responsive element; ABRE: Abscisic Acid Response Element; TGACG-motif: Jasmonate-responsive element; P-box: Gibberellin-responsive element; GARE-motif: Gibberellic Acid Response Element; TC-rich repeats: Defense/stress-responsive element; LTR: Low-Temperature Responsive Element; ARE: Anaerobic Response Element; GC-motif: Anoxic specific enhancer; MBS: MYB Binding Site; WUN-motif: Wound-responsive Wound-responsive element
