# Supplementary material for: Genome-Wide Identification, Characterization, and Expression Analysis of NRT Gene Family in Suaeda glauca
Source: Biology (Basel). 2025 Aug 21;14(8):1097. doi: 10.3390/biology14081097 (PMC12383926; doi:10.3390/biology14081097)
Supplement: Supplementary file 1 [file biology-14-01097-s001.zip › Supplemental Figures.pdf]

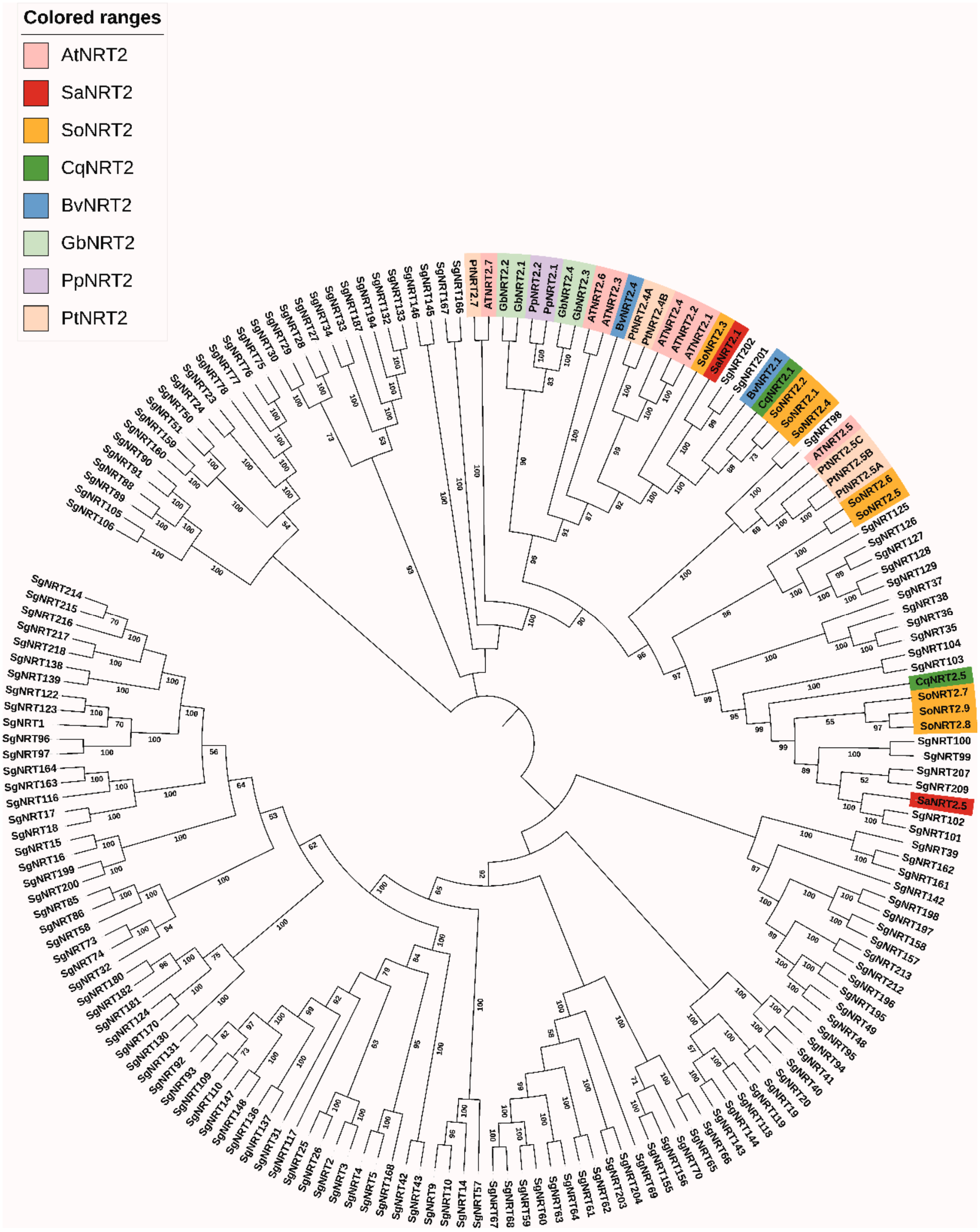

Figure S1. The phylogenetic tree of *Suaeda glauca* NRT2 and NRT2 members from other species. A total of 26 sequences from seven species, including *Ginkgo biloba*, *Populus trichocarpa*, *Pinus pinaster*, *Suaeda altissima*, *Chenopodium quinoa*, *Beta vulgaris*, and *Spinacia oleracea*, were selected to construct the phylogenetic tree with SgNRT2s. Bootstrap values are shown in the figure.

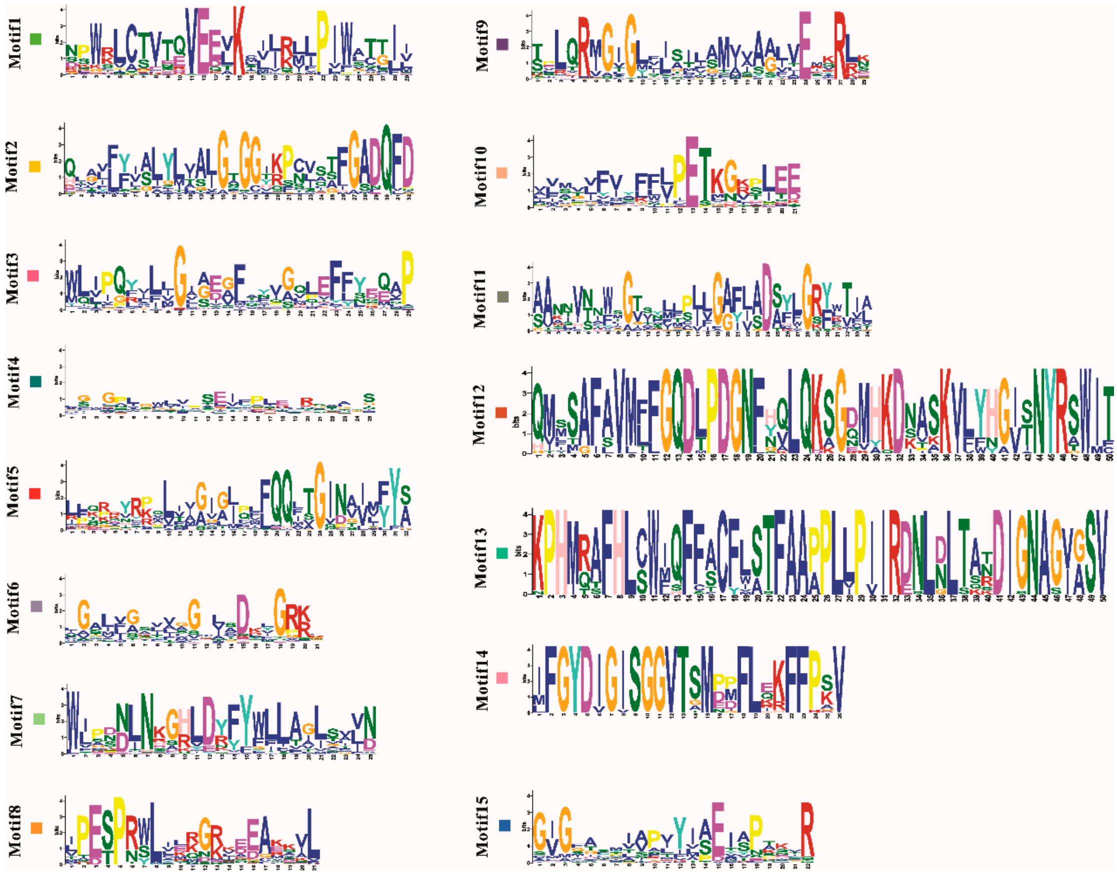

Figure S2. Logos of 15 conserved motifs in *Suaeda glauca*. Logos representing motifs conserved within SgNRT1 and SgNRT2 clades. The 15 differently colored squares correspond to the legends in Figure 2A.
